# Bibliometric Review to Explore Emerging High-Intensity Interval Training in Health Promotion: A New Century Picture

**DOI:** 10.3389/fpubh.2021.697633

**Published:** 2021-07-23

**Authors:** Yanwei You, Wenkai Li, Jianxiu Liu, Xingtian Li, Yingyao Fu, Xindong Ma

**Affiliations:** ^1^Division of Sport Science & Physical Education, Tsinghua University, Beijing, China; ^2^China Table Tennis College, Shanghai University of Sport, Shanghai, China

**Keywords:** high intensity interval training, health promotion, bibliometric analysis, CiteSpace, VOSviewer

## Abstract

**Background:** High-intensity interval training (HIIT) is an emerging exercise strategy and is considered to be a recipe for health promotion. This study aimed to systematically identify collaboration networks, track research trends, highlight current hotspots, and predict future frontiers in HIIT and its applications in health promotion since the start of the new century.

**Methods:** Relevant original publications were obtained from the Science Citation Index Expanded of the Web of Science Core Collection (WoSCC) database between 2001 and 2020. CiteSpace and VOSviewer software were used to perform bibliometric visualization and comparative analysis of involved indexes that included countries, institutions, journals, authors, references, and keywords.

**Results:** A total of 572 papers were included, and the trend of annual publications showed a remarkable growth. The United States and the University of Exeter were the most productive country and institutions, respectively, with 107 and 18 publications, respectively. *European Journal of Applied Physiology* took the lead in the number of published articles, and *Medicine and Science in Sports and Exercise* ranked first in the cocitation counts. Barker AR and Gibala MJ were considered as the most productive and the most highly-cited authors.

**Conclusions:** “Health risks,” “adolescent,” and “aging” are the three noteworthy topics during the evolution of HIIT-health promotion (HIIT-HP) research. The current research hotspots of HIIT and its practices in the health promotion domain lies in “metabolic diseases,” “cardiovascular diseases,” “neurological diseases,” and “musculoskeletal diseases.” The authors summarize that “prevention and rehabilitation,” “micro and molecular level,” and “cognition and mental health” are becoming frontiers and focus on the health topics related to HIIT in the upcoming years, which are worthy of further exploration.

## Introduction

The rising prevalence of several chronic diseases (1–5) [e.g., obesity, type 2 diabetes (T2D), and hypertension] worldwide has been described as global puzzles and public health problems (6–8). The relationship between exercise and health promotion has been gradually explored and studied. In 2007, the American College of Sports Medicine and the American Medical Association colaunched a health promotion program named “exercise is medicine” (9). High-intensity interval training (HIIT), in various forms, is an emerging training method that generally refers to repeated sessions of relatively brief intermittent exercise, often performed with an “all-out” effort or at an intensity close to VO_2_ peak (10–12). Billat defined HIIT as a method that involves repeated short-to-long bouts of high-intensity exercise interspersed with recovery periods (13). With the transition and development of the basic science of physiology, a series of major advancements in exercise training has spurred in the field of sports science, and among them, HIIT is one of the most effective training methods. Many studies have shown that HIIT can enhance cardiorespiratory and metabolic function and, in turn, the fitness of the athletes (13–15). Prior evidence also showed that a sufficient volume of HIIT can increase peak oxygen uptake (VO_2_ peak) and the maximal activity of mitochondrial enzymes in skeletal muscles (16, 17). In contrast to other training techniques, such as moderate-intensity continuous training (MICT) and resistance training (RT), HIIT is a time-efficient, universally applicable, and enjoyable strategy. In recent years, HIIT is not only used for elite athletes training but is also considered as an exercise prescription or complementary therapy for the rehabilitation of related chronic diseases. Therefore, it has been significantly developed and widely applied to the field of exercise science and health promotion over the last 20 years.

Scholars have conducted a number of reviews (18–22) on various aspects of HIIT in different contexts, but some defects still needed to be improved: (i) most reviews used the meta-based methodology, although it can support the effects of HIIT from the evidence-based medicine level, while this type of review cannot provide an overview of all applications of HIIT in the fields of health promotion; (ii) the samples in some systematic reviews were mainly based on subjective screening, and the sample size was rather small; (iii) the included research objects were not comprehensive (such as only a review of randomized controlled trials) or were concentrated only on specific and limited aspects.

Bibliometric is a quantitative analysis strategy that involves mathematics and statistics to identify publications on a particular topic (23, 24). Based on comprehensive indexes like journals, authors, countries, and institutions, it can conduct an in-depth evaluation of the research trends and the focus of a certain field (25–27). Previous studies have shown that tracing knowledge diffusions and using cluster analysis can provide a rounded overview in several interdisciplinary research (28–30). Moreover, the results of the bibliometric evaluation can provide suggestions for future research and decision-making. CiteSpace is a java-based scientific mapping software developed by Chen (Drexel University, Philadelphia, PA, USA) for bibliometric and comparative analysis (31). VOSviewer is another scientometric tool for creating maps based on network data and for visualizing and exploring these maps. By presenting numerous data in the form of knowledge maps, the productivity of authors and institutions, geographic distributions, and cooperative relations can reflect the development of a discipline and research tendency. CiteSpace and VOSviewer have been recently applied in various fields, such as medical treatment, cognitive function, and pain management (32–34).

To the best of our knowledge, studies on the health benefits of HIIT have sprung up over the past two decades, whereas a few of them are engaged in collecting global data, evaluating the emerging trends, and conducting reviews of HIIT–health promotion (HIIT-HP) fields from the perspective of visualization and bibliometric analysis. Hence, the cooperative network, research trend, current status, and future frontier in this field are unknown and urgently need to be explored. This is the first study that pioneers the use of bibliometric strategy into the investigation of the HIIT-HP domain. This study is anticipated to help researchers extract hidden information for further studies in exercise and health-related fields and provide them with valuable guidance in selecting frontier topics by answering the following central questions:

(i) Which countries, institutions, journals, authors, and references lead the research trend in global HIIT-HP fields?
(ii) What are the current hotspots and major domains that HIIT can be used in health promotion nowadays?
(iii) Where are the frontiers and perspectives of related health topics in the future of HIIT?

## Materials and Methods

### Data Acquisition and Search Strategy

All research materials were retrieved from the Science Citation Index Expanded (SCI-E) of the WoSCC database on May 15, 2021. We completed the search within the same day to avoid any bias caused by database updates. The reason for using this database is that SCI-Expanded in WoSCC contains comprehensive citation index records, which includes numerous influential and high-quality journals all over the world. Additionally, a previous study indicated that Web of Science had better accuracy than Scopus and some other databases (35). Therefore, it is a comprehensive and authoritative database for literature mining, especially in the field of natural science (36).

**Figure 1:**
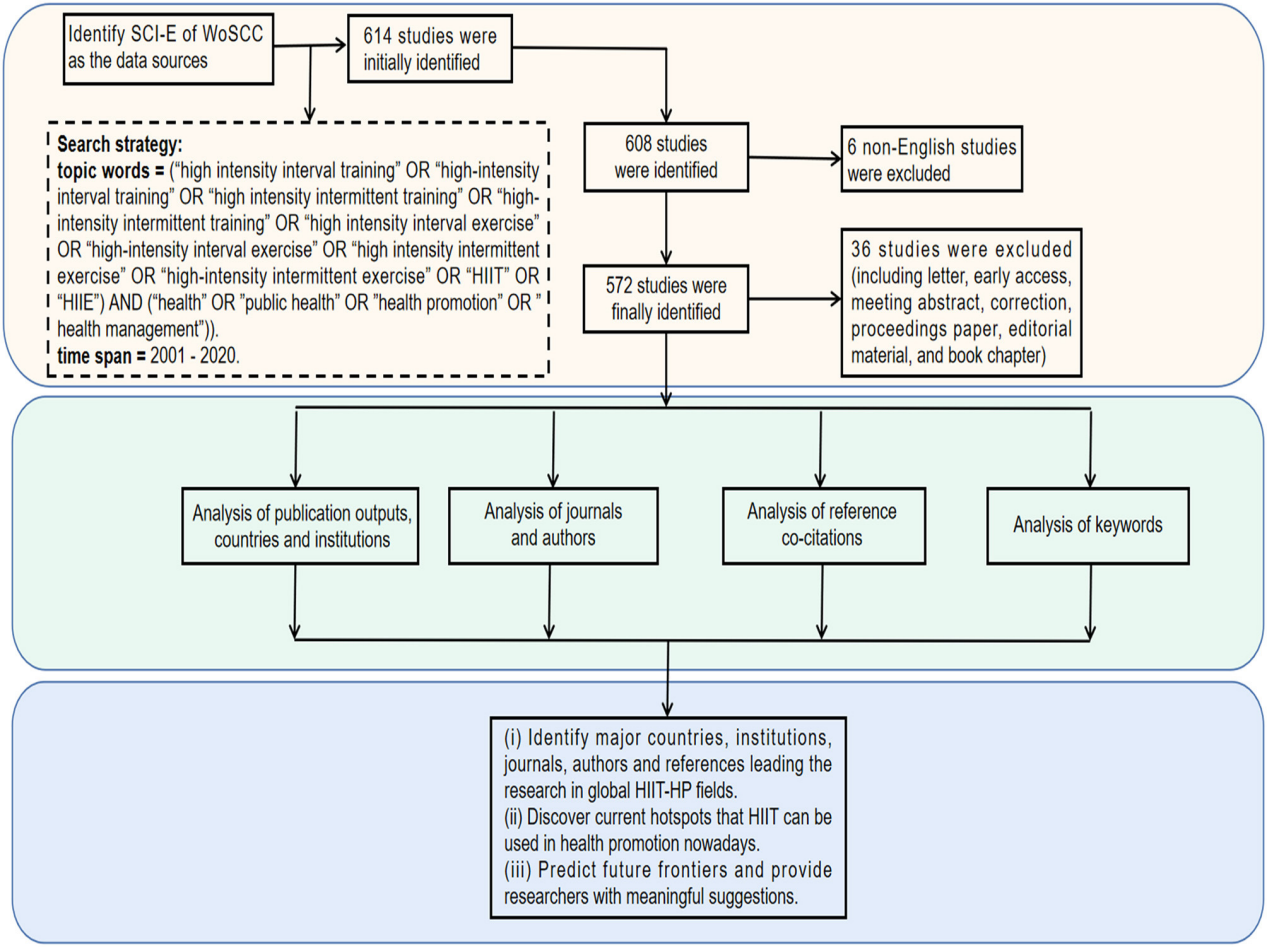
The summary of the flowchart and study design.

The following methods were conducted for search publications: topic words = [(“high intensity interval training” OR “high-intensity interval training” OR “high intensity intermittent training” OR “high-intensity intermittent training” OR “high intensity interval exercise” OR “high-intensity interval exercise” OR “high intensity intermittent exercise” OR “high-intensity intermittent exercise” OR “HIIT” OR “HIIE”) AND (“health” OR “public health” OR “health promotion” OR “health management”)], time span = 2001–2020. Referring to the previous bibliometric studies (37, 38), we only selected “articles or reviews” for analysis, and the language was limited to “English”; other document types and non-English articles were excluded. Basic information for each research was gathered into text documents, such as countries, institutions, journal sources, authors, and references. The detailed search strategy and inclusion criteria in this study are summarized in Figure 1.

### Analysis Tool

CiteSpace and VOSviewer were used to conduct statistical analyses on the literature. This analysis drew a series of progressive visualization knowledge domains to detect emerging trends, hidden implications, and landmark literature. Parameters consisting of publication amount, impact factor, centrality, and occurrence/citation burst were applied. Impact factor (IF) is recognized as an international standard criterion for evaluating the impact of a journal (39), and the impact factors in this review are based on the Journal Citation Reports (2019). Centrality is an index for measuring the importance of a node in a network, and a node with a large size typically indicates high occurrence or citation frequency as a pivotal point (31, 40, 41). Occurrence burst denotes a term that frequently occurs over a given period, which can be considered as research hotspots (41, 42). Microsoft Excel 2016 software was applied to describe and predict the publication trend of HIIT researches. The function model was set as follows: f(*x*) = a*x*^3^ + b*x*^2^ + c*x* + d, in which *x* represented the year of publication and f(*x*) demonstrated the cumulative amount of publications. The reason for using a cubic polynomial function instead of linear, logarithmic, or other functions was that it can effectively and accurately capture information about publication trends (43). The reason for not applying the exponential function was to prevent the occurrence of no publication in potential years. Through the methods mentioned above, we can have a profound understanding of the research history, current status, and future prediction of HIIT studies in the health promotion field.

## Results

### Analysis of Publication Outputs

**Figure 2:**
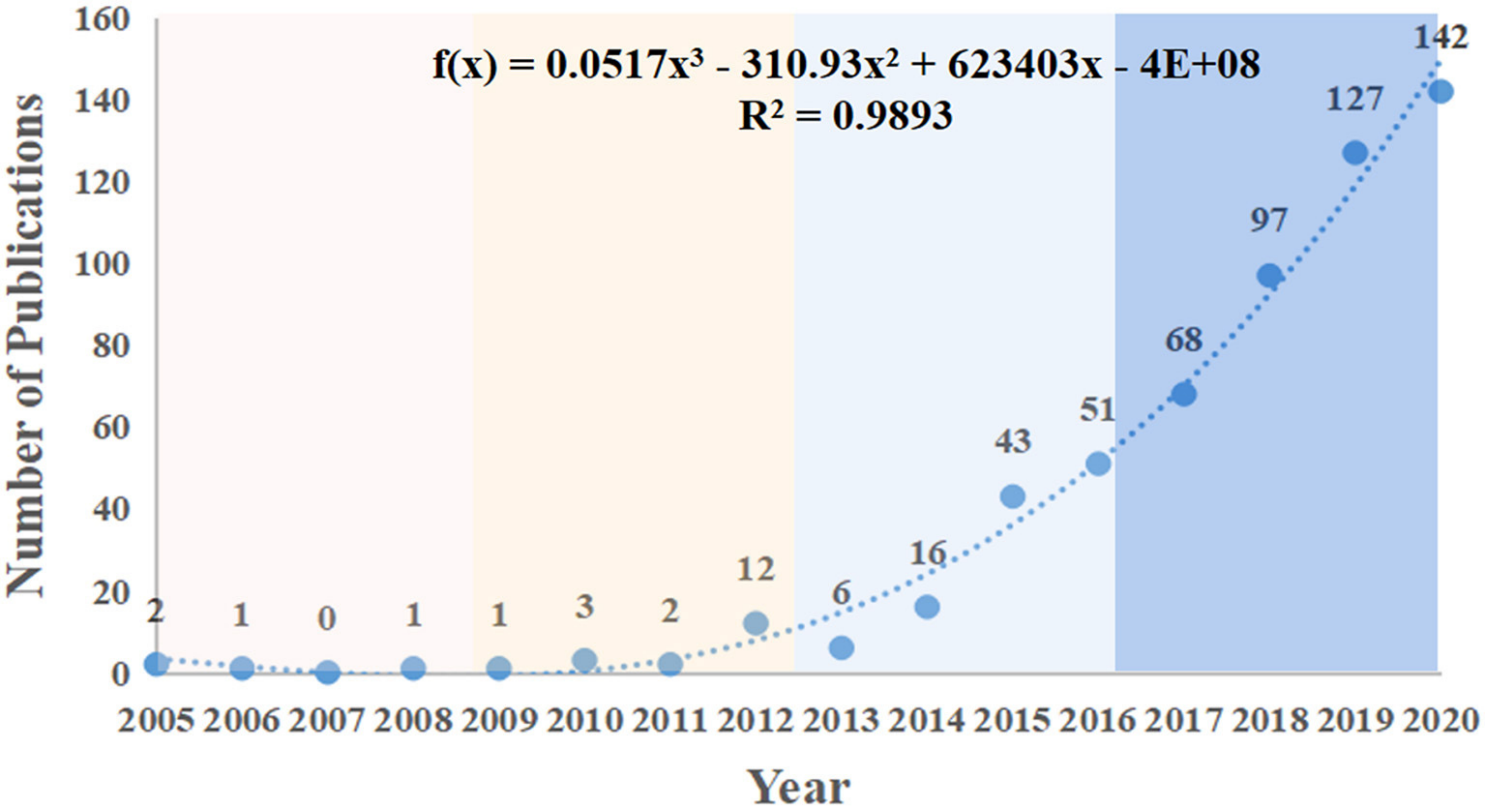
The output of publications and growth trends of HIIT in health promotion fields.

Since there was no publication before 2004, we started the calculation at the beginning of 2005. A total of 572 records (including 494 articles and 78 reviews) satisfied the search criteria and were used for further analysis. The relationship between the number of articles per year and the development tendency on HIIT research in the health promotion field is shown in Figure 2. The overall trend of publications increased from 2 in 2005 to 142 in 2020. The number of publications on HIIT has increased remarkably within the last two decades, and the growth trend model (*R*^2^ = 0.9893) demonstrates that more research on the applications of HIIT in the health promotion domain is ongoing and that this field seems to be progressive and promising.

Publications on HIIT can be divided into three stages: the initial stage (2005–2012), the second stage (2012–2016), and the third stage (2016–2020). Before 2012, the annual publication remained at a low level, while it exceeded 10 for the first time in 2012. After that, the amount of literature published gradually showed a continuous upward trend and broke through 50 for the first time in 2016. From the perspective of annual publications, we consider the years 2012 and 2016 to be the critical turning points. In terms of the development of HIIT, in 1920, a famous runner named Paavo Nurmi had already used HIIT in his training routines (12). After that, many scholars contributed to the popularization of this specific training format in the previous century. However, their publications were not widely recognized and promoted. In the early 2000s, Laursen and Jenkins (16) reviewed the scientific basis for HIIT and suggested that the optimal HIIT program intensity, duration, and recovery needed further exploration, which pointed out the direction for future HIIT research. In 2012, Gibala et al. (44) indicated that HIIT can not only be applied to train athletes but can also served as an effective alternative way to traditional endurance-based training in preventing health burdens associated with a couple of chronic diseases. This discovery prompted the application of HIIT intervention in the prevention and rehabilitation of chronic diseases, which also contributed to the advancement and application of HIIT technology.

### Analysis of Countries and Institutions

**Figure 3:**
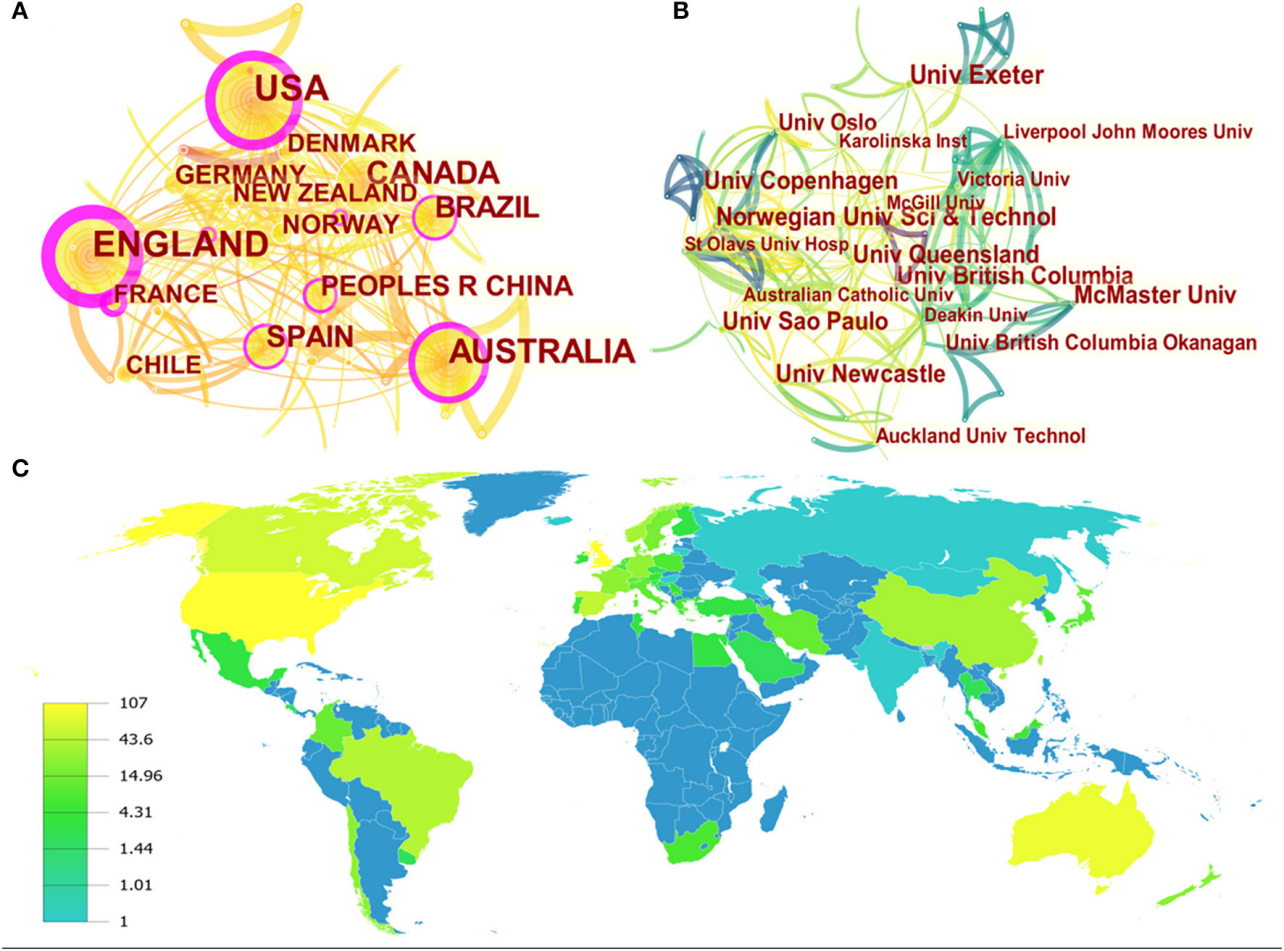
The map of countries **(A)**, institutions **(B)**, and world distribution **(C)** of publications in the HIIT-HP field.

A total of 56 countries/regions were involved in HIIT and its applications in the health promotion domain. The details of the top 10 countries and institutions are presented in Table 1. The United States and the United Kingdom were in the dominant positions, with 107 and 106 publications, respectively. Australia was the third productive country and published 88 literatures totally, followed by Canada (63 publications) and Spain (51 publications). The contributions by the top four countries were all above 50, which indicated that they made major contributions in research achievements. As shown in Figure 3A, the collaborations among these countries were generated by CiteSpace. In the matter of centrality (the purple outer ring of the circle), the wider the circle, the higher is the centrality. According to the definition of centrality (31), the countries shown on the map had close collaborations with each other and produced tremendous academic influence.

**Table 1:** Ranking of top 10 countries and institutions involved in the HIIT-HP domain.

**Rank**	**Country**	**Publications**	**Centrality**	**Institution**	**Publications**	**Centrality**
1	United States	107	0.51	University of Exeter	18	0.03
2	United Kingdom	106	0.68	Norwegian University of Science and Technology	16	0.08
3	Australia	88	0.20	University of British Columbia	15	0.06
4	Canada	63	0.02	University of Copenhagen	15	0.06
5	Spain	51	0.14	University of Newcastle	15	0.02
6	Brazil	43	0.11	McMaster University	14	0.01
7	China	35	0.11	University of Queensland	14	0.04
8	Norway	33	0.04	University of Saõ Paulo	14	0.06
9	Germany	26	0.08	University of Granada	11	0.01
10	France	24	0.25	University of Oslo	11	0.01

A total of 270 institutions participated in the application of HIIT in the health promotion field. As listed in Table 1, the University of Exeter was the leading institution in the quantity of published works (18 publications), followed by the Norwegian University of Science and Technology (16 publications), the University of British Columbia (15 publications), the University of Copenhagen (15 publications), and the University of Newcastle (15 publications). The network map of institution cooperation was generated using CiteSpace (Figure 3B). In terms of centrality, the Norwegian University of Science and Technology (0.08) was the first-tier institution. Besides that, the University of British Columbia, the University of Copenhagen, and the University of Saõ Paulo shared the same centrality (0.06) and took the lead in cooperation as well. Additionally, the distribution of the countries and regions participating in HIIT-HP research all over the world is displayed in Figure 3C, and the scholars of this field were mainly distributed in North America, Europe, and Asia.

### Analysis of Journals and Co-cited Journals

A total of 234 scholarly journals published articles on the application of HIIT in the health fields. As shown in Table 2, the top 10 journals accounted for almost a third of the total publications, *European Journal of Applied Physiology* (IF 2019 = 2.580) published the highest amount of articles (26 publications, 4.55%), followed by *Frontiers in Physiology* and *Medicine and Science in Sports and Exercise*. Compared with other journals, the contributions of the top three journals were all above 20 publications and 4% proportions, thus signifying that they had a special position in HIIT-HP fields. Among the 10 journals with the largest amount of literature published, the average IF of these 10 journals was 2.718, whereas the impact factors from only three journals exceeded 3.0. It seemed that publishing HIIT-related topics in a high-impact-factor journal could not be an easy task.

**Table 2:** Ranking of top 10 journals and co-cited journals involved in the HIIT-HP domain.

**Rank**	**Journal**	**Publications**	**Percentage (%)**	**IF (2019)**	**Cited journal**	**Co-citation counts**
1	European Journal of Applied Physiology	26	4.55	2.580	Medicine and Science in Sports and Exercise	464
2	Frontiers in Physiology	24	4.20	3.367	Sports Medicine	364
3	Medicine and Science in Sports and Exercise	24	4.20	4.029	Plos One	346
4	International Journal of Environmental Research and Public Health	19	3.32	2.849	Journal of Applied Physiology	333
5	Plos One	18	3.15	2.740	Journal of Physiology-London	290
6	Journal of Sports Sciences	17	2.97	2.597	Circulation	283
7	Applied Physiology Nutrition and Metabolism	16	2.80	2.522	European Journal of Applied Physiology	272
8	Scandinavian Journal of Medicine Science in Sports	16	2.80	3.255	British Journal of Sports Medicine	252
9	Journal of Sports Medicine and Physical Fitness	12	2.10	1.432	Applied Physiology Nutrition and Metabolism	204
10	Journal of Sports Science and Medicine	11	1.92	1.806	JAMA-Journal of the American Medical Association	175

**Figure 4:**
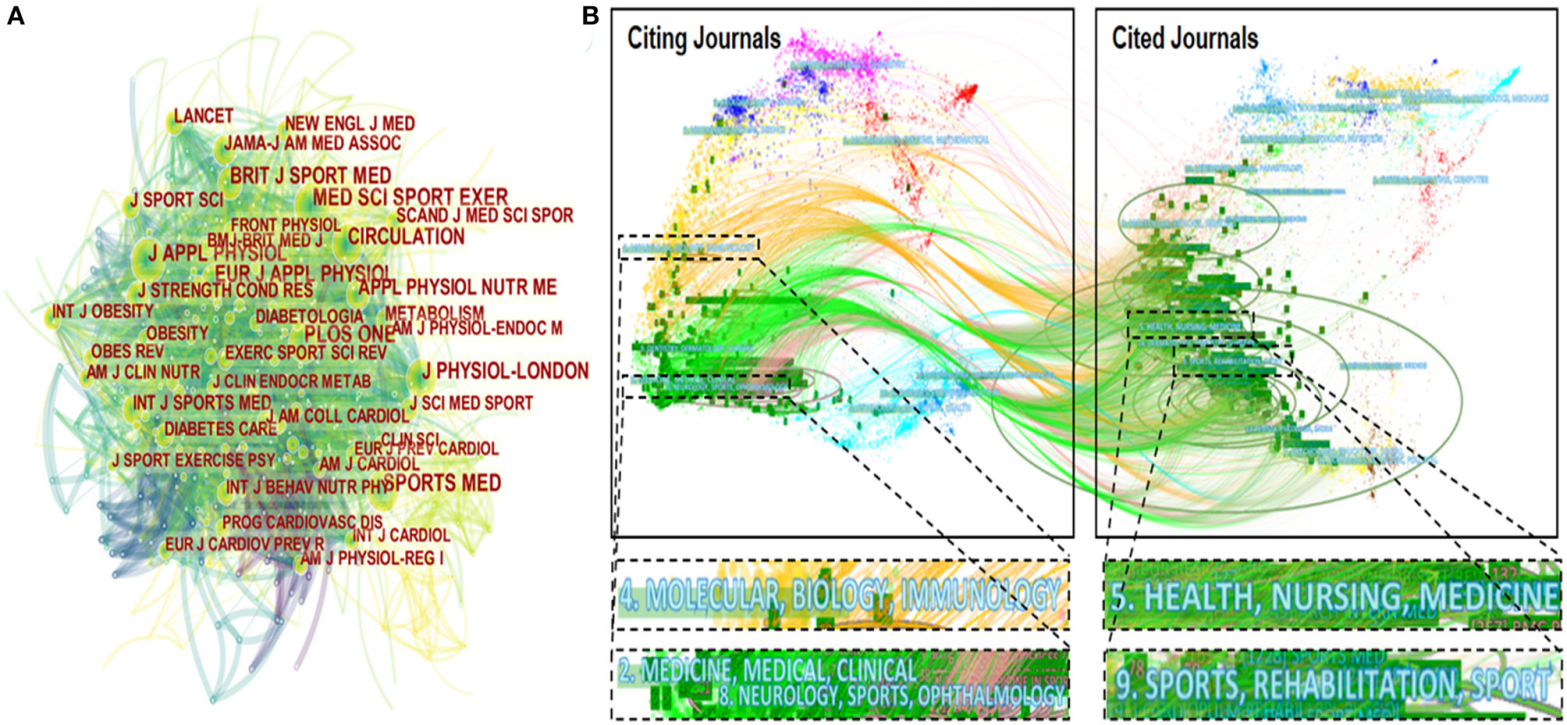
The map of co-cited journals **(A)**; a dual-map overlay of journals that published literature in the HIIT-HP field **(B)**.

In cited journal list, *Medicine and Science in Sports and Exercise* ranked first with 464 co-citation counts, *Sports Medicine* and *PLOS ONE* also contributed 364 and 346, respectively. The high citation counts, to some extent, can illustrate that these journals had academic competitiveness and were considered mainstreams in the HIIT-HP field. The network map of cited journals was plotted by CiteSpace with 538 nodes and 3,724 links. Figure 4A presents that the distribution of the focus of the periodical publication is extremely extensive, including sports science, physiology, rehabilitation medicine, public health, and several comprehensive interdisciplinary research. As the cited journals offer a theoretical basis for the citing journals, these diverse trajectories indicate that the disciplinary center of the journals moved from a single subject to multidisciplinary clusters.

Figure 4B shows a dual-map overlay of journals that published literature related to HIIT-HP fields. This approach uses two graphs at the same time. On the left are the categories of citing journals, and on the right are the disciplines of cited journals. Citation links can display the ins and outs of citations data sets. As the citing journals put forward views on the basis of the cited journals, this map can reflect the citation relationship between disciplines to some extent. In general, there are mainly two citation paths, the light orange path presents that literature published in “Molecular, Biology, Immunology” journals preferred to quote journals mostly in the fields of “Health, Nursing, Medicine.” The grass green path shows that articles published in “Medicine, Medical, Clinical” and “Neurology, Sports, Ophthalmology” journals tended to cite journals primarily in the “Sports, Rehabilitation, Sport” domain.

### Analysis of Authors and Co-cited Authors

Three hundred and sixty-six authors contributed to the total number of papers. The top 10 authors involved in HIIT-HP research are shown in Table 3. These authors have published a total of 81 papers, accounting for 14.16% of all published papers on HIIT-HP research. Barker AR and Williams CA ranked first concerning publication output (12 publications), followed by Jung ME and Little JP with 9 publications. The top five co-cited authors were Gibala MJ, Wisloff U, Tjonna AE, Little JP, and Gillen JB. These authors were active authors in the field of HIIT-HP research.

**Table 3:** Ranking of top 10 authors, co-cited authors, and co-cited references in the HIIT-HP domain.

**Rank**	**Author**	**Counts**	**Co-cited author**	**Counts**	**Co-cited reference**	**Counts**
1	Barker AR	12	Gibala MJ	206	Weston KS, 2014, BRIT J SPORT MED, V48, P1227	75
2	Williams CA	12	Wisloff U	131	Gibala MJ, 2012, J PHYSIOL-LONDON, V590, P1077	62
3	Jung ME	9	Tjonna AE	113	Biddle SJH, 2015, INT J BEHAV NUTR PHY, V12, P0	56
4	Little JP	9	Little JP	110	Ramos JS, 2015, SPORTS MED, V45, P679	50
5	Eather N	8	Gillen JB	110	Costigan SA, 2015, BRIT J SPORT MED, V49, P0	47
6	Lubans DR	7	Weston KS	103	Milanovic Z, 2015, SPORTS MED, V45, P1339	47
7	Castillo MJ	6	Burgomaster KA	88	Weston M, 2014, SPORTS MED, V44, P1005	45
8	Delao A	6	Cohen J	74	Batacan RB, 2017, BRIT J SPORT MED, V51, P0	45
9	Ramirezcampillo R	6	Jung ME	68	Gillen JB, 2014, APPL PHYSIOL NUTR ME, V39, P409	38
10	Amarogahete FJ	6	Garber CE	66	Jung ME, 2014, PLOS ONE, V9, P0	37

**Figure 5:**
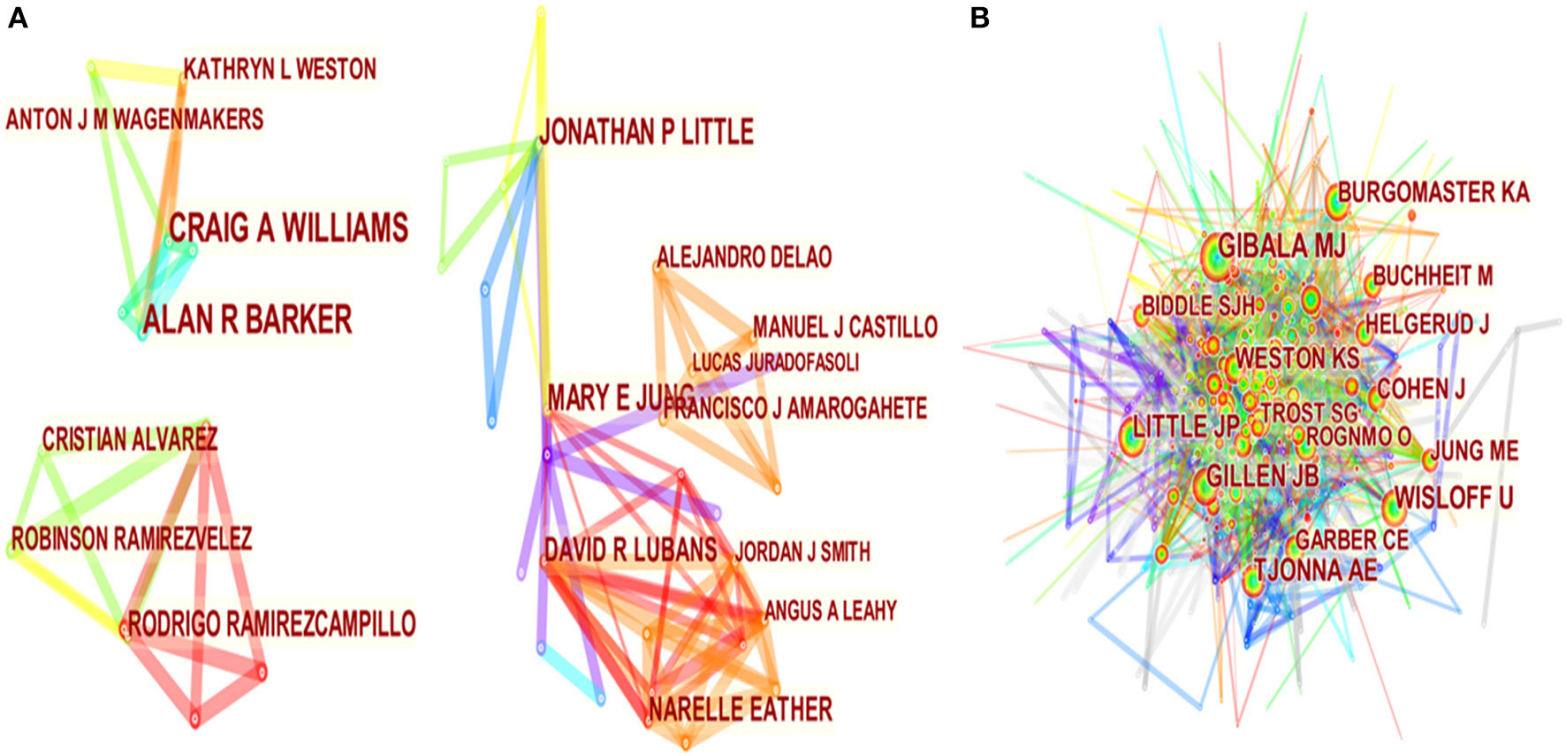
The map of cooperation network of the authors in the HIIT-HP field **(A)**; the map of the connections of the co-cited authors in the HIIT-HP field **(B)**.

Table 3 presents the top 10 most co-cited references as well. These references prepared the ground and accelerated the development of research in the HIIT-HP field. The top co-cited reference was authored by Weston KS and was published in *the British Journal of Sports Medicine* (one of the most influential journals in sports medicine), which reported the effects of HIIT in patients with lifestyle-induced cardio-metabolic disease (45). The co-cited count of articles ranked 2–8 also exceeds 40, which to some extent hints that the achievements of these research studies in this field are highly recognized. Cooperation between authors and co-cited authors is analyzed for the identification of potential partnerships. Connections represent cooperation relationships among nodes, and the thickness of the connection represents the closeness of cooperation. The merged network of authors in Figure 5A is composed of 366 nodes and 696 links. In this figure, the cooperation network of the author is further divided into three smaller clusters, which are scattered and led by several superstar authors. Figure 5B illustrates the connections between co-cited authors (nodes = 558, links = 3,288). The knowledge maps of the authors of these papers and co-cited authors can offer crucial information on influential research groups and potential partners, enabling researchers to establish cooperation.

### Analysis of Reference Co-citation

**Figure 6:**
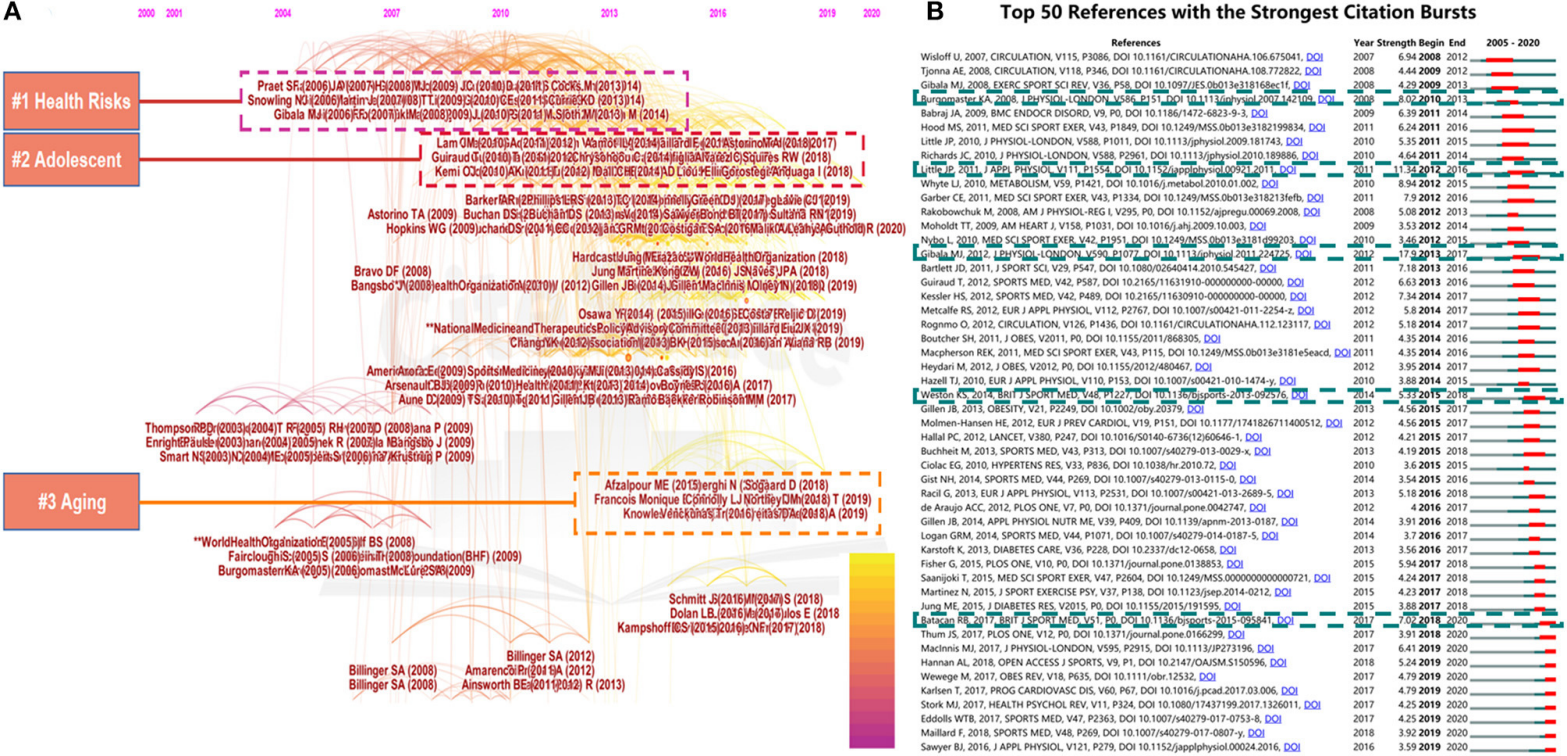
The timeline view map based on the reference co-citation analysis in the HIIT-HP field **(A)**; top 50 references with the strongest citation burst in the HIIT-HP field **(B)**.

Reference co-citation analysis is one of the significant indices in bibliometric studies, which is usually applied to explore research focuses in a given academic field (46). Articles and their co-citation relevant data were used to create major clusters, and then knowledge domains of these clusters were constructed by analyzing reference co-citation. All kinds of research related to the HIIT-HP field between 2000 and 2020 were grouped into a number of major clusters. Each cluster was associated with the citation index, achievement field, and critical literature series within a period, making a grand spectacle of distinct specialty or thematic concentration. The timeline view for the major clusters, which illustrates the time interval and research advance in the progress and evolution of the subdomain of each cluster, is shown in Figure 6A. In CiteSpace, the modularity value (*Q-*value) and the weighted mean silhouette value (*S*-value) are regarded as the evaluation strategies to assess the standard of clustering, and a mean of *Q* > 0.5 and *S* > 0.7 implies that the cluster is convincing. In Figure 6A, the value of *Q* equals 0.6382 and that of S equals 0.8141, which further verifies the rationality of this clustering strategy.

We used the log-likelihood ratio (LLR) strategy to generate reference co-citation clustering, with the type of selection using “Keywords Option,” since the creator of CiteSpace software suggested that the LLR algorithm performed best for covering the “uniqueness and coverage” of labels and keywords detection and can accurately present the classification of all clusters. Here, we introduced the following three noteworthy topics, “Health Risks,” “Adolescent,” and “Aging,” which have been of wide concern recently and would be discussed in depth in the discussion part.

References with the strongest citation burst are considered as the research basics of frontiers in the upcoming years (47, 48). As shown in Figure 6B, the top 50 references were identified in terms of their strongest citation bursts. Among these references, Gibala et al. (44) provided insights on the utility of HIIT for improving physiological adaptations in health and disease and highlighted suggestions for future research. Little et al. (49) focused on investigating the role of HIIT in reducing hyperglycemia and increasing muscle mitochondrial volume in patients with T2D. Burgomaster et al. (50) analyzed and compared the similar metabolic adaptations of humans during the low-volume sprint interval and traditional endurance training from a physiological point of view. In recent years, high burst articles showed that HIIT had a positive role in treating chronic diseases caused by an unhealthy lifestyle. Two reviews focusing on the effectiveness of HIIT in the treatment of cardiovascular-related disease published in *the British Journal of Sports Medicine* by Batacan et al. (51) and Weston et al. (45) with burst strength as 7.02 and 5.33 represented a strong research hotspot.

### Analysis of Keywords

**Figure 7:**
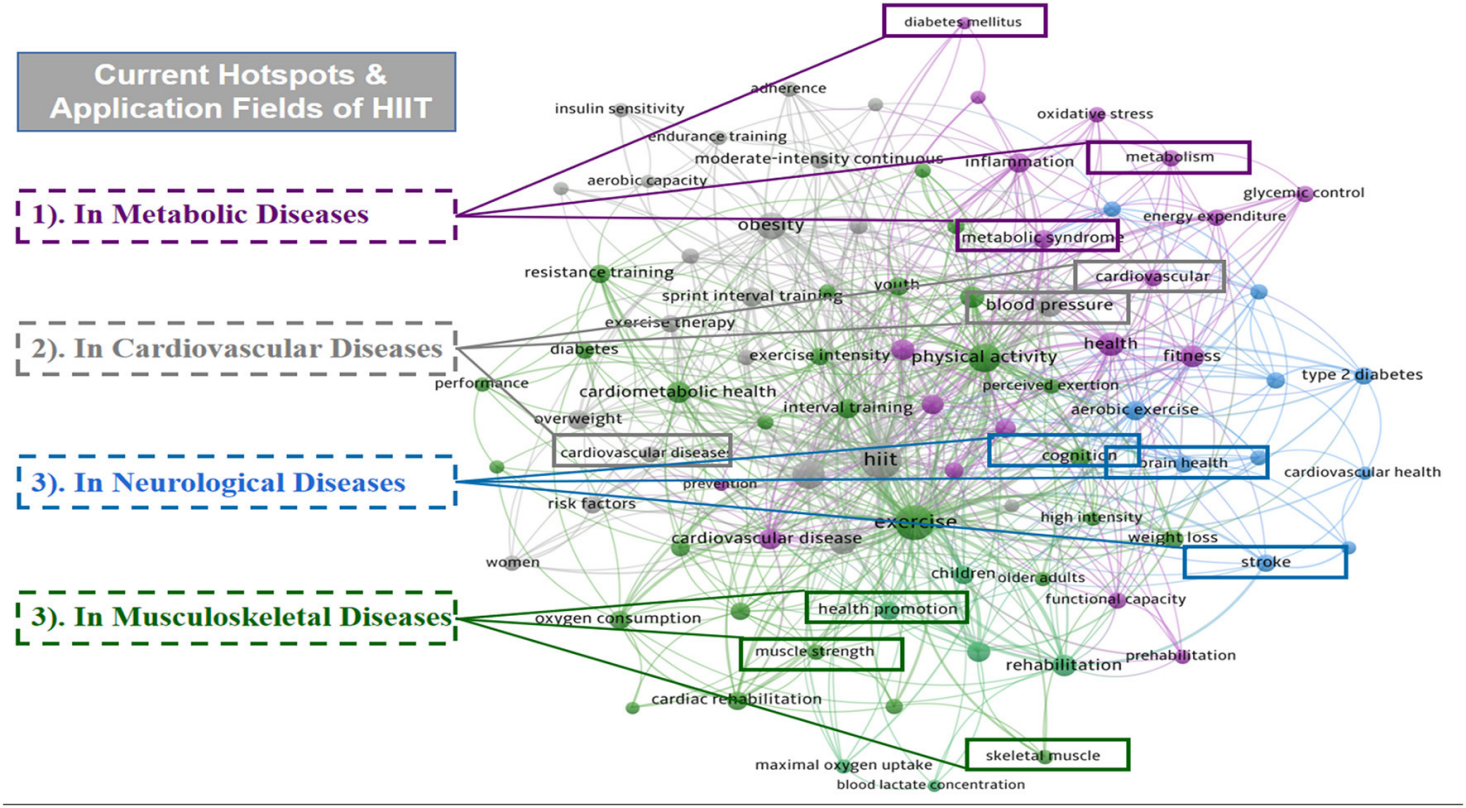
The distribution of the co-occurrence network of keywords in the HIIT-HP field.

A map of keywords can present major objects and hot topics of research. VOSviewer software was applied to conduct the co-occurrence network of keywords in this study. We created the map on the basis of bibliographic data with a full counting strategy, setting the minimum number of occurrences of a keyword as four. By using a thesaurus to clean and purify the data, 85 keywords out of the total 1,163 keywords met the threshold and are presented in Figure 7. According to the research categories of keywords, we integrated the classification generated by VOSviewer algorithm and further divided all these keywords into several major clusters with four different colors. In this figure, violet purple, light gray, photo blue, and grass green are represented for the applications of HIIT in “Metabolic Diseases,” “Cardiovascular Diseases,” “Neurological Diseases,” and “Musculoskeletal Diseases,” respectively. These application domains would be further discussed in the next part.

**Figure 8:**
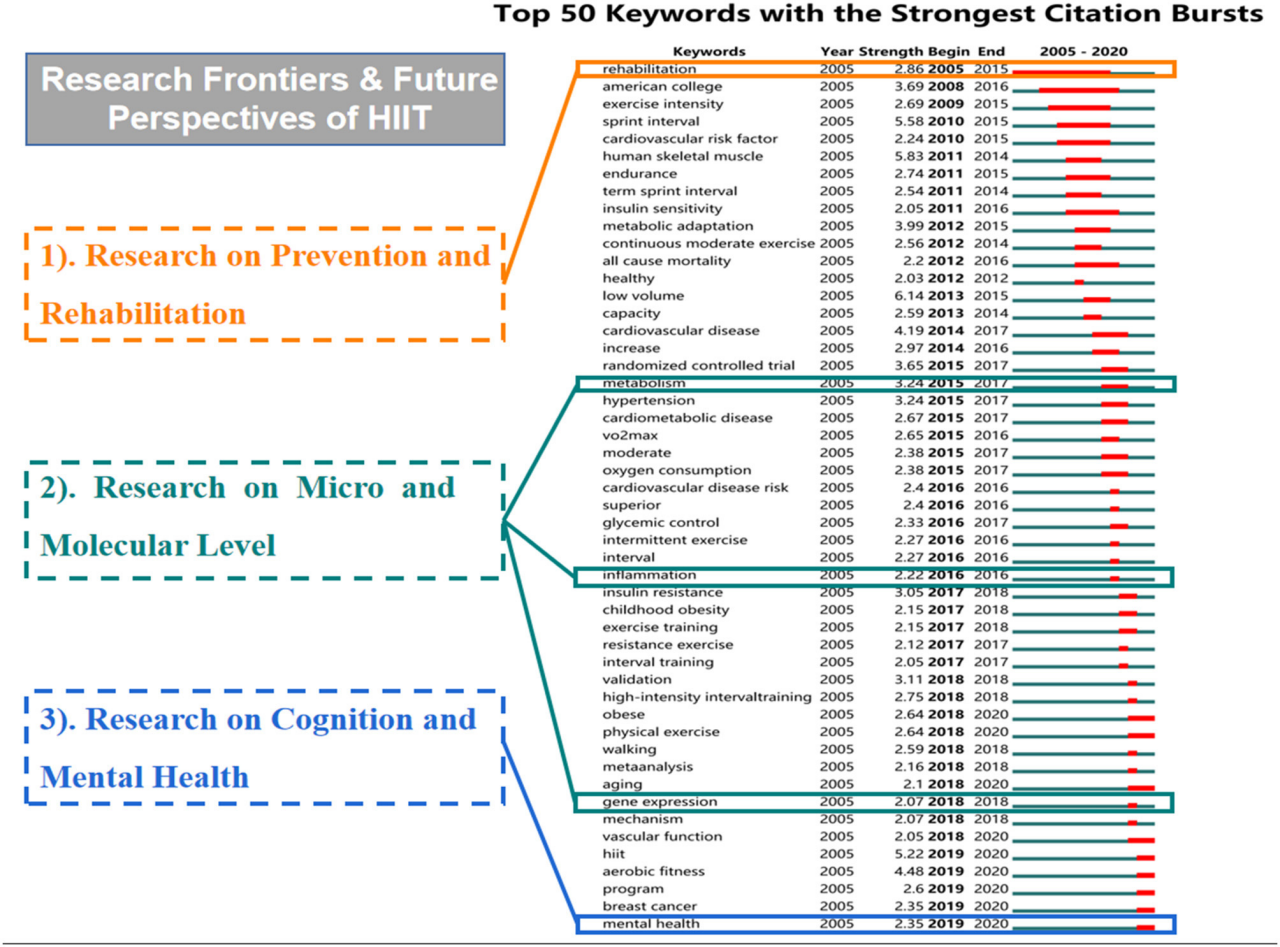
Top 50 keywords with the strongest citation burst in the HIIT-HP field.

The top five keywords with the highest citation burst in HIIT-HP research were low volume (6.14), human skeletal muscle (5.83), sprint interval (5.58), HIIT (5.22), and aerobic fitness (4.48). The values in brackets reflect the strength of burst. Occurrence burst, which indicates the steep increment of a keyword in appearance over a period of time, can reflect development in frontier topics and dynamics in a research field (41, 42, 52). The top 50 keywords with the strongest occurrence burst are shown in Figure 8. In the first decade of the twenty-first century, critical physiological indexes such as skeletal muscle, cardiovascular capacity, respiratory function, and other physical performances induced by HIIT were widely mentioned and studied. Around the year 2015, with the physiological mechanisms of HIIT gradually been recognized, it seemed to be a research trend to apply HIIT to the rehabilitation of chronic diseases. Burst keywords like “heart disease,” “cardiovascular disease,” “obesity,” and other health-related problems were dominant in this period. From 2018 to 2020, keywords with the strongest citation bursts were focusing on the molecular level and mental benefits of HIIT recently, including “gene expression” and “mental health.” This result verifies that “prevention and rehabilitation,” “micro and molecular level,” and “cognition and mental health” are becoming frontiers and focuses in HIIT-HP fields, which are worthy of further exploration.

## Discussion

High-intensity interval training has become one of the most popular training methods for different groups of people to keep fit all over the world. In country and institution analysis, information about regional distribution presented that HIIT-HP researches are currently ongoing worldwide. Among the top 10 prolific countries, eight are developed countries and only two are developing countries. This implies that developed countries may invest more resources in the field of sports science and medicine. People living in developed regions usually pay more attention to fitness and wellness, so they also have a relatively high quality of life and a longer life span. However, developing countries, such as China, tended to catch up in the field of exercise and health science, especially in the HIIT-HP domain. Moreover, a previous study reported that the United States, China, and Brazil were also among the most productive countries in diabetes-related publications (53). We suppose that a large population base and aging tendency may lead to an increase in health risks, which tends to be an important driving force for these countries to invest resources in health researches.

Discovery and exploration of remarkable authors, references, and journals were carried out in this study. Authors from different regions and institutions who participated in the HIIT research made noteworthy contributions to studies in this field. Figures 5A,B show that the relationship between co-cited authors seems stronger, while the authors have cooperation only in part and lack communication and contact in general. Under the background of interdisciplinary integration, more scholars from different fields should strengthen mutual communication and cooperation and jointly promote the research progress and application of the HIIT method. As shown in Figures 4A,B, the network map and dual-map overlay of journals demonstrate several relevant publishing sources in the HIIT-HP field. Sports science-related journals were still the primary source to publish HIIT research. Physiology, rehabilitation or preventive medicine, and public health journals had a certain interest in this field as well. In addition, comprehensive journals also played an important role in publishing HIIT articles.

On the basis of the timeline view map of reference co-citation analysis, we could disclose research evolution trends. From Figures 6A,B, three high-frequency topics and clusters were summarized and analyzed. “Health Risks,” “Adolescent,” and “Aging” are three noteworthy topics during the development of HIIT-HP research:

(i) For health risks and factors: In the early stage of HIIT-HP study, scholars mostly focused on the risk factors of health-related problems. The two main factors that cause health risks are excessive energy intake and physical inactivity (54–57). The former gives rise to overweight or obesity risks, and the latter often leads to a sedentary lifestyle. It is almost universally acknowledged that obesity leads to multiple health risks (55, 57). Member states of the WHO appealed for a voluntary target to stop the rise in obesity by 2025, and exercise has been considered as widespread call to counteract overweight in all populations. Compared with MICT or other training methods, HIIT has its unique advantages in treating overweight people. A number of recent research have already proved that HIIT favored better body composition improvements and expended less time to achieve similar effects than MICT in curing obesity (58, 59). When it comes to problems related to a sedentary lifestyle, converging epidemiological evidence demonstrates that a sedentary lifestyle may lead to incompatible chronic diseases (54, 56). From an evolutionary point of view, the adaptation that HIIT-related exercise brings to the body is an excellent strategy against the lifestyle-induced evolutionary mismatch (45, 51).
(ii) For youth and adolescent groups: In the initial development of HIIT, its applications were mostly promoted among young athletes (16, 60). With the development of exercise-training theory, HIIT is no longer a unique recipe for elite athletes, and children and teenagers also have access to this method to keep healthy. Adolescence is a critical period for physical growth and development. In view of the fact that teenagers are still in the growth stage, all-extremity HIIT will have a long-term impact on shaping their cardiopulmonary endurance, skeletal muscle, and circulatory system. The most common model used in HIIT research is the Wingate Test, which asks teenagers to complete an “all out” cycling session of 30 s against high-resistance on a specialized cycle ergometer. After a typical training session with four to six repeats interspersed by 4 min of recovery, the oxidative capacity of skeletal muscle of the youth groups and the maximum enzyme activity or protein content of mitochondria can be improved (61). In addition, metabolic abnormalities and obesity-related problems caused by academic stress, lack of sleep, and a sedentary lifestyle are concerning points in the development of teenagers. Some emerging researches have supported that HIIT can efficiently improve cardio-metabolic capacity in overweight teenagers compared with traditional endurance training (62–64). While HIIT has already been praised in teenagers for its beneficial effects on body composition and cardio-metabolic health, its application to improve cognitive and mental health also has great research potential. One study using neuroimaging methods like functional near-IR spectroscopy detected that acute HIIT can strengthen the cognitive and executive functions of teenagers by increasing the activation of the task-related area of the brain prefrontal cortex (65). In summary, it is promising to promote the application of HIIT in the mind–body shaping of teenagers.
(iii) For elderly groups: The sharp increase of the aging population has become a public issue currently (66, 67). Aging is associated with decreased aerobic fitness, cardiac remodeling, and bone-density loss, which may lead to an increased risk of chronic diseases. It is well-acknowledged that exercise may have the potential to delay the aging process, while weight-bearing activities are frequently limited due to musculoskeletal and balancing problems in the elderly groups. HIIT can be considered as a suitable supplement of strength training method in the elderly, and studies have shown that HIIT can improve the physical performance of the elderly group (68–70). In particular, when referring to the specific practicing strategy, the elderly are recommended to perform HIIT step by step. For example, when first using this method, one can practice for 20 s and rest for 30 s, and several weeks later, one can elevate the HIIT mode to an upgraded level (e.g., vigorous training for 30 s and resting for 15 s). It is better to avoid fatigue of the same muscle group (e.g., high resistance dumbbell bending), and the effective method is to combine HIIT with low-impact movements to reduce the impact on the joints and soft tissues. Additionally, it is worth noting that HIIT plays an important role in the health promotion of post-menopausal women who are more special in the elderly group. Numerous studies focusing on women suggested an increased risk of metabolic diseases in midlife (71, 72), and the management of fitness and weight at the menopause stage is becoming another public health concern (73). Several research studies (74–76) indicated that HIIT can improve health indicators, including inflammatory and adipokine profiles, in post-menopausal women. A recent meta-analysis (77) also showed that cycling HIIT seemed to be better than running in post-menopausal women, and interventions over 8 weeks with three sessions per week should be recognized as a promoted method.

On the basis of the co-occurrence map of keywords, we could not only explore current hotspots but also explore the applications of HIIT in several health promotion fields. From Figure 7, a number of prevalent keywords and noteworthy domains of HIIT-HP research were concluded and analyzed. Here, we further analyzed the following four categories based on the application fields of the HIIT program:

(i) Metabolic diseases: Diabetes, especially T2D and hyperglycemia, is regarded as one of the most common metabolic illnesses (78, 79). In 2014, Francois and his colleagues (80) tried to examine the effects of brief bouts of exercise performed before meals (“exercise snacks”) in the treatment of post-prandial hyperglycemia in individuals with T2D. It did appear that HIIT may lead to greater reductions in post-prandial hyperglycemia when compared with moderate-intensity continuous exercise (80–82). Besides, different studies targeting multiple groups also indicated that HIIT is beneficial for the rehabilitation of T2D problems in a number of perspectives (83–85). One recent review using meta-regression concluded that the mechanism of HIIT for treating T2D lied in reducing both insulin resistance and body weight (86). However, the application of HIIT in the treatment of type 1 diabetes seems less, and the metabolism in preventing and managing other associated cardiovascular complications are still worthy of further study.
(ii) Cardiovascular diseases: Blood pressure is closely correlated with cardiovascular health, and hypertension has led to high cardiovascular morbidity and mortality worldwide (87–89). Current guidelines (90, 91) recommend a moderate-intensity approach for most inactive adults with pre-hypertension to established hypertension. However, several studies have found that HIIT provided comparable reductions in resting blood pressure and was associated with greater improvements in dealing with hypertension when compared to MICT (92, 93). More specifically, in lifestyle-induced cardio-metabolic diseases, the mechanism of the benefits of HIIT may due to its central and peripheral adaptations to the body (45). To summarize, exercise prescriptions like HIIT, as well as other health care methods, play a crucial role in achieving blood pressure control in patients with circulation disorders by reinforcing their cardiorespiratory fitness and cultivating healthy lifestyle habits.
(iii) Neurological diseases: Based on the neuromuscular and proprioceptive benefits, HIIT can also be used as counter measures for some neurological diseases. Taking stroke as an example, it has been ranked as the second most common cause of death worldwide from the global burden of diseases, injuries, and risk factors study (94). Previous guidelines suggested that MICT in aerobics can be used as the strategy for improving mobility, aerobic capacity, and cardiovascular health in stroke groups (95, 96). However, recent evidence indicated that HIIT might be more effective than MICT for both aerobic and motor outcomes in these patients (97–99). Furthermore, it was reported that the innate ability of the nervous system to repair itself from damage through neuroplasticity formed the basis of stroke rehabilitation (100, 101). The rationality of using HIIT may be due to its strength to enhance cognitive functions, including upregulating the neuroplasticity markers in the hippocampus and cortex (102–104). HIIT has been proved as a feasible and promising strategy in individuals with stroke symptoms, and further HIIT studies are ongoing in patients with other brain diseases such as Alzheimer's disease and multiple sclerosis.
(iv) Musculoskeletal diseases: Skeletal muscles account for almost half of the human body mass and play essential roles in maintaining health. HIIT program can also be used to prevent sarcopenia, a more common syndrome in middle-aged and elderly patients, which is often combined with muscle mass loss, oxidative stress, chronic low-grade inflammatory status, and adipocytokine dysfunction (105, 106). Both animal and human experiments (106–108) showed that HIIT could modulate changes in inflammatory parameters, adipose tissues, and serum, as well as insulin-like growth factor 1 (IGF-1) levels in the skeletal muscle tissue. One recent research (109) also proved that HIIT was an effective training strategy to improve sarcopenia and skeletal muscle vascularization compared with traditional resistance training.

The detection of the burst keywords, referred to currently, can be used to explore the future development tendency. Hence, by analyzing these burst keywords (Figure 8) in the HIIT-HP field, we summarized the following three topics, which are considered as frontiers and expected to frequently occur over the coming years and signify research trends:

(i) Research on the prevention and rehabilitation: Based on our discussion of current hotspots and applications mentioned above, we can preliminarily find that HIIT can help to prevent or treat health burdens associated with many chronic diseases. Considering that preventive medicine is increasingly emphasized and valued nowadays, exercise intervention plays an important and irreplaceable role in this area. As a highly efficient, multi-stimulated and cost-saving means of exercise intervention, HIIT will possess broad prospects in the field of medicine and rehabilitation under the fast-paced and time-insufficient social background. Moreover, HIIT is characterized by short bursts or vigorous activity, interspersed by periods of rest or low-intensity exercise for recovery (110). So it has almost infinite forms and variables, and the specific physiological performance induced by this form of training is determined by multiple factors, including intensity, duration time, quantity of intervals performed, and activity patterns during recovery. The training performance of different modes and exercise stimuli in diverse subjects is still worthy of investigation in the future.
(ii) Research on micro-level and molecular level: With the development of life science technology, the current literature can further explain the healthy improvements conducted by HIIT from the molecular level. Previous studies revealed that aerobic-related adaptations of HIIT can include an incremental capacity for musculoskeletal lipid oxidation and rising metabolic transport proteins (50, 61, 111), which are beneficial to the mitigation and rehabilitation of specific diseases. The imbalance between pro- and anti-inflammatory cytokines can lead to diseases related to immunity and metabolism disorders (112, 113). Exercise can influence the immune system by changing the phenotype of leukocytes and producing more skeletal muscle-derived interleukin (IL)-6 and IL-10 (114–116). Studies have suggested longer duration exercises can make these improvements, but what about a short but intensive training program? New findings showed that short-term training like HIIT can also reduce the ability of IL-10 to LPS-induced TNF-α production (117). Furthermore, the metabolic crosstalk between these tissues and the molecular mechanism of these changes are still unclear, and more gene expressions, regulatory factors, and circulatory pathways need to be discovered to prove the systemic adaptations induced by HIIT.
(iii) Research on cognition and mental health: Recently, some researches on psychology and mental health have made further elaboration on the advantages of HIIT from the spiritual level. The increase in awareness of “active health” of people and the change in their attitudes toward HIIT are reinfluencing their perception of this fitness model. As terrible mental health conditions of modern people (such as anxiety, depression, and even suicidal tendencies) increase, research using HIIT strategy to counter depressive symptoms or other mood disorders are explored (118–121). Exercise can trigger the secretion of multiple hormones in the brain, such as brain-derived neurotrophic factor (BDNF, related to memory and information transmission), dopamine (DA, related to attention and satisfaction), norepinephrine (NE, related to complex emotions and motivational awakening), and serotonin (5-HT, which is related to depression, anxiety, impulsivity, and other emotions). Therefore, HIIT can enhance focus and reduce impulsive thoughts, which can mitigate a number of mental and emotional disorders. While HIIT seems to be difficult for beginners, it could evoke experiences of incompetence, failure, and lower self-esteem. Thus, the optimal HIIT regimen and the novel training mode aimed at solving such mental puzzles remain undefined and need more exploration.

Noticeably, under the context of the coronavirus disease 2019 (COVID-19) pandemic (122), isolation may lead to less exercise and more health-related problems. Although outdoor games are typically more available, varied, and usually have more infrastructures, HIIT has its unique advantage that can be performed and completed independently at home in such a special period. With the COVID-19 pandemic, the world faced unprecedented medical challenges. Many of the infected patients suffered from severe respiratory impairment, neurasthenia, and cardiac disability due to the virus. However, evidence has suggested that HIIT may be a potential strategy for cardiac rehabilitation therapy in patients with the COVID-19 infection, which would be beneficial to improving the quality of life and long-term prognosis (123). Moreover, HIIT has similar or even superior effects on a range of physiological performance, immune system, and health-related markers of both healthy individuals and diseased populations.

This review has some limitations. First, in order to meet the data format standard of visualization tools like CiteSpace and VOSviewer, we only extracted data from the WoSCC. However, the SCI-E of the WoSCC database is one of the most extensive and well-recognized global resources, which has been used in several previous high-quality bibliometric studies. Second, this study generated a language- and article-type bias since we only considered “English” literature published as “articles or reviews,” although “English” remains the most common language for publishing academic documents worldwide, and “articles or reviews” are the mainstream types of publications. Last, but not least, the greater amount of publications or cooperation networks are not always more important or inspired. Readers are recommended to focus on both the quality of researches and their citation status if they want to follow certain topics or hotspots.

There are multiple strengths of this research. It is one of the pioneering studies to tackle the specific HIIT topic in health science. Visualization software is used in analyzing the data, which allowed for a more holistic exploration of the literature with appropriate figures and tables. Moreover, the study is closely relevant to the scope of the journal, and it would be beneficial to an audience of freshmen, professional scholars, fitness coaches, doctors, rehabilitation therapists, and any other targeted readers. We sincerely hope that this bibliometric review of global publications can provide an in-depth and broad perspective to advance and expand the applications of HIIT in numerous public health promotion affairs.

## Conclusion

This study applied a bibliometric and comparative analysis and provided a new century picture for HIIT and its applications in the health promotions field. We offered both a historical and a prospective insight into HIIT strategy and provided information for researchers regarding potential cooperative relationships, popular issues, development trends, and frontier topics. The number of published articles, crucial countries and institutions, published journals, authors and co-cited authors, and cooperative networks were systematically analyzed using hybrid analysis and visualization technologies (Q1). As an emerging exercise intervention to help people maintain health, HIIT has received increasing attention. During the development of HIIT-HP project, “health risks,” “adolescent,” and “aging” are three noteworthy research objects. We suggest that the current research hotspots of HIIT and its practices in the health promotion domain are “metabolic diseases,” “cardiovascular diseases,” “neurological diseases,” and “musculoskeletal diseases” (Q2). In addition, “prevention and rehabilitation,” “micro and molecular level,” and “cognition and mental health” are becoming frontiers and hotspots (Q3), which should receive more attention in the future.

## Data Availability Statement

The original contributions presented in the study are included in the article/Supplementary Material, further inquiries can be directed to the corresponding author/s.

## Author Contributions

YY, WL, and JL: conceptualization and writing—review and editing. YY, XL, and YF: methodology. YY and JL: validation and data curation. YY, JL, XL, and YF: formal analysis. YY: writing—original draft preparation. XM: supervision, project administration, and funding acquisition. All authors have read and agreed to the published version of the manuscript.

## Conflict of Interest

The authors declare that the research was conducted in the absence of any commercial or financial relationships that could be construed as a potential conflict of interest.

## Publisher's Note

All claims expressed in this article are solely those of the authors and do not necessarily represent those of their affiliated organizations, or those of the publisher, the editors and the reviewers. Any product that may be evaluated in this article, or claim that may be made by its manufacturer, is not guaranteed or endorsed by the publisher.

## References

[1] Finucane MM Stevens GA Cowan MJ Danaei G Lin JK Paciorek CJ. National, regional, and global trends in body-mass index since 1980: systematic analysis of health examination surveys and epidemiological studies with 960 country-years and 9.1 million participants. Lancet. (2011) 377:557–67. 10.1016/S0140-6736(10)62037-5 21295846 PMC4472365

[2] Poulter NR Prabhakaran D Caulfield M. Hypertension. Lancet. (2015) 386:801–12. 10.1016/S0140-6736(14)61468-9 25832858

[3] Zheng Y Ley SH Hu FB. Global aetiology and epidemiology of type 2 diabetes mellitus and its complications. Nat Rev Endocrinol. (2018) 14:88–98. 10.1038/nrendo.2017.151 29219149

[4] de Onis M Blossner M Borghi E. Global prevalence and trends of overweight and obesity among preschool children. Am J Clin Nutr. (2010) 92:1257–64. 10.3945/ajcn.2010.29786 20861173

[5] Fielding RA Vellas B Evans WJ Bhasin S Morley JE Newman AB. Sarcopenia: an undiagnosed condition in older adults. Current consensus definition: prevalence, etiology, and consequences. International working group on sarcopenia. J Am Med Dir Assoc. (2011) 12:249–56. 10.1016/j.jamda.2011.01.003 21527165 PMC3377163

[6] Ginter E Simko V. Type 2 diabetes mellitus, pandemic in 21st century. Adv Exp Med Biol. (2012) 771:42–50. 10.1007/978-1-4614-5441-0_6 23393670

[7] Qamar A Braunwald E. Treatment of hypertension: addressing a global health problem. JAMA. (2018) 320:1751–2. 10.1001/jama.2018.16579 30398610

[8] Swinburn BA Sacks G Hall KD McPherson K Finegood DT Moodie ML. The global obesity pandemic: shaped by global drivers and local environments. Lancet. (2011) 378:804–14. 10.1016/S0140-6736(11)60813-1 21872749

[9] Sallis RE. Exercise is medicine and physicians need to prescribe it! Br J Sports Med. (2009) 43:3–4. 10.1136/bjsm.2008.054825 18971243

[10] Gibala MJ McGee SL. Metabolic adaptations to short-term high-intensity interval training: a little pain for a lot of gain? Exerc Sport Sci Rev. (2008) 36:58–63. 10.1097/JES.0b013e318168ec1f 18362686

[11] Gibala MJ. High-intensity interval training: a time-efficient strategy for health promotion? Curr Sports Med Rep. (2007) 6:211–3. 10.1007/s11932-007-0033-8 17617995

[12] Bartlett JD Close GL MacLaren DP Gregson W Drust B Morton JP. High-intensity interval running is perceived to be more enjoyable than moderate-intensity continuous exercise: implications for exercise adherence. J Sports Sci. (2011) 29:547–53. 10.1080/02640414.2010.545427 21360405

[13] Billat LV. Interval training for performance: a scientific and empirical practice. Special recommendations for middle- and long-distance running. Part I: aerobic interval training. Sports Med. (2001) 31:13–31. 10.2165/00007256-200131010-00002 11219499

[14] Billat LV. Interval training for performance: a scientific and empirical practice. Special recommendations for middle- and long-distance running. Part II: anaerobic interval training. Sports Med. (2001) 31:75–90. 10.2165/00007256-200131020-00001 11227980

[15] Esfarjani F Laursen PB. Manipulating high-intensity interval training: effects on VO2max, the lactate threshold and 3000 m running performance in moderately trained males. J Sci Med Sport. (2007) 10:27–35. 10.1016/j.jsams.2006.05.014 16876479

[16] Laursen PB Jenkins DG. The scientific basis for high-intensity interval training: optimising training programmes and maximising performance in highly trained endurance athletes. Sports Med. (2002) 32:53–73. 10.2165/00007256-200232010-00003 11772161

[17] Ross A Leveritt M. Long-term metabolic and skeletal muscle adaptations to short-sprint training: implications for sprint training and tapering. Sports Med. (2001) 31:1063–82. 10.2165/00007256-200131150-00003 11735686

[18] Rugbeer N Constantinou D Torres G. Comparison of high-intensity training versus moderate-intensity continuous training on cardiorespiratory fitness and body fat percentage in persons with overweight or obesity: a systematic review and meta-analysis of randomized controlled trials. J Phys Act Health. (2021) 18:610–23. 10.1123/jpah.2020-0335 33837165

[19] Sa Filho AS Cheniaux E de Paula CC Murillo-Rodriguez E Teixeira D Monteiro D. Exercise is medicine: a new perspective for health promotion in bipolar disorder. Expert Rev Neurother. (2020) 20:1099–107. 10.1080/14737175.2020.1807329 32762382

[20] Leal JM Galliano LM Del Vecchio FB. Effectiveness of high-intensity interval training versus moderate-intensity continuous training in hypertensive patients: a systematic review and meta-analysis. Curr Hypertens Rep. (2020) 22:26. 10.1007/s11906-020-1030-z 32125550

[21] Cao M Quan M Zhuang J. Effect of high-intensity interval training versus moderate-intensity continuous training on cardiorespiratory fitness in children and adolescents: a meta-analysis. Int J Environ Res Public Health. (2019) 16:1533. 10.3390/ijerph16091533 31052205 PMC6539300

[22] Logan GR Harris N Duncan S Schofield G. A review of adolescent high-intensity interval training. Sports Med. (2014) 44:1071–85. 10.1007/s40279-014-0187-5 24743929

[23] Oelrich B Peters R Jung K. A bibliometric evaluation of publications in urological journals among European Union countries between 2000-2005. Eur Urol. (2007) 52:1238–48. 10.1016/j.eururo.2007.06.050 17673361

[24] Bornmann L Leydesdorff L. Scientometrics in a changing research landscape: bibliometrics has become an integral part of research quality evaluation and has been changing the practice of research. EMBO Rep. (2014) 15:1228–32. 10.15252/embr.201439608 25389037 PMC4264924

[25] Ellegaard O Wallin JA. The bibliometric analysis of scholarly production: how great is the impact? Scientometrics. (2015) 105:1809–31. 10.1007/s11192-015-1645-z 26594073 PMC4643120

[26] Aydinoglu AU Taskin Z. Origins of life research: a bibliometric approach. Orig Life Evol Biosph. (2018) 48:55–71. 10.1007/s11084-017-9543-4 28702783

[27] Kim HJ Yoon DY Kim ES Lee K Bae JS Lee JH. The 100 most-cited articles in neuroimaging: a bibliometric analysis. Neuroimage. (2016) 139:149–56. 10.1016/j.neuroimage.2016.06.029 27327516

[28] Yu DJ Sheng LB. Knowledge diffusion paths of blockchain domain: the main path analysis. Scientometrics. (2020) 125:471–97. 10.1007/s11192-020-03650-y

[29] Yu DJ Pan TX. Tracing knowledge diffusion of TOPSIS: a historical perspective from citation network. Expert Syst Appl. (2021) 168:114238. 10.1016/j.eswa.2020.114238

[30] Yu DJ Chen YT. Dynamic structure and knowledge diffusion trajectory research in green supply chain. J Intell Fuzzy Syst. (2021) 40:4979–91. 10.3233/JIFS-201720

[31] Chen CM. CiteSpace II: detecting and visualizing emerging trends and transient patterns in scientific literature. J Am Soc Inf Sci Tec. (2006) 57:359–77. 10.1002/asi.20317

[32] Liao HC Tang M Luo L Li CY Chiclana F Zeng XJ. A bibliometric analysis and visualization of medical big data research. Sustainability-Basel. (2018) 10:166. 10.3390/su1001016628034409

[33] Zheng K Wang X. Publications on the association between cognitive function and pain from 2000 to 2018: a bibliometric analysis using citespace. Med Sci Monit. (2019) 25:8940–51. 10.12659/MSM.917742 31762442 PMC6894366

[34] Weng LM Zheng YL Peng MS Chang TT Wu B Wang XQ. A bibliometric analysis of nonspecific low back pain research. Pain Res Manag. (2020) 2020:5396734. 10.1155/2020/5396734 32215136 PMC7085391

[35] Yeung AWK. Comparison between Scopus, Web of Science, PubMed and publishers for mislabelled review papers. Curr Sci India. (2019) 116:1909–14. 10.18520/cs/v116/i11/1909-1914

[36] Yi F Yang P Sheng H. Tracing the scientific outputs in the field of Ebola research based on publications in the Web of Science. BMC Res Notes. (2016) 9:221. 10.1186/s13104-016-2026-2 27083891 PMC4832479

[37] Yan W Zheng K Weng L Chen C Kiartivich S Jiang X. Bibliometric evaluation of 2000-2019 publications on functional near-infrared spectroscopy. Neuroimage. (2020) 220:117121. 10.1016/j.neuroimage.2020.117121 32619709

[38] Lu C Li X Yang K. Trends in shared decision-making studies from 2009 to 2018: a bibliometric analysis. Front Public Health. (2019) 7:384. 10.3389/fpubh.2019.00384 31921749 PMC6930165

[39] Smith MJ Weinberger C Bruna EM Allesina S. The scientific impact of nations: journal placement and citation performance. PLoS ONE. (2014) 9:e109195. 10.1371/journal.pone.0109195 25296039 PMC4189927

[40] Liang YD Li Y Zhao J Wang XY Zhu HZ Chen XH. Study of acupuncture for low back pain in recent 20 years: a bibliometric analysis via CiteSpace. J Pain Res. (2017) 10:951–64. 10.2147/JPR.S132808 28479858 PMC5411170

[41] Chen C Hu Z Liu S Tseng H. Emerging trends in regenerative medicine: a scientometric analysis in CiteSpace. Expert Opin Biol Ther. (2012) 12:593–608. 10.1517/14712598.2012.674507 22443895

[42] Chen CM Dubin R Kim MC. Orphan drugs and rare diseases: a scientometric review (2000-2014). Expert Opin Orphan Drugs. (2014) 2:709–24. 10.1517/21678707.2014.920251

[43] Qi B Jin S Qian H Zou Y. Bibliometric analysis of chronic traumatic encephalopathy research from 1999 to 2019. Int J Environ Res Public Health. (2020) 17:5411. 10.3390/ijerph17155411 32731338 PMC7432826

[44] Gibala MJ Little JP MacDonald MJ Hawley JA. Physiological adaptations to low-volume, high-intensity interval training in health and disease. J Physiol-Lond. (2012) 590:1077–84. 10.1113/jphysiol.2011.224725 22289907 PMC3381816

[45] Weston KS Wisloff U Coombes JS. High-intensity interval training in patients with lifestyle-induced cardiometabolic disease: a systematic review and meta-analysis. Br J Sports Med. (2014) 48:1227–34. 10.1136/bjsports-2013-092576 24144531

[46] Trujillo CM Long TM. Document co-citation analysis to enhance transdisciplinary research. Sci Adv. (2018) 4:e1701130. 10.1126/sciadv.1701130 29308433 PMC5752411

[47] Synnestvedt MB Chen C Holmes JH. CiteSpace II: visualization and knowledge discovery in bibliographic databases. AMIA Annu Symp Proc. (2005) 2005:724–8. 16779135 PMC1560567

[48] Fitzpatrick RB. Essential science indicators. Med Ref Serv Q. (2005) 24:67–78. 10.1300/J115v24n04_05 16203702

[49] Little JP Gillen JB Percival ME Safdar A Tarnopolsky MA Punthakee Z. Low-volume high-intensity interval training reduces hyperglycemia and increases muscle mitochondrial capacity in patients with type 2 diabetes. J Appl Physiol. (2011) 111:1554–60. 10.1152/japplphysiol.00921.2011 21868679

[50] Burgomaster KA Howarth KR Phillips SM Rakobowchuk M Macdonald MJ McGee SL. Similar metabolic adaptations during exercise after low volume sprint interval and traditional endurance training in humans. J Physiol. (2008) 586:151–60. 10.1113/jphysiol.2007.142109 17991697 PMC2375551

[51] Batacan RB Jr. Duncan MJ Dalbo VJ Tucker PS Fenning AS. Effects of high-intensity interval training on cardiometabolic health: a systematic review and meta-analysis of intervention studies. Br J Sports Med. (2017) 51:494–503. 10.1136/bjsports-2015-095841 27797726

[52] Kleinberg J. Bursty and hierarchical structure in streams. Data Min Knowl Disc. (2003) 7:373–97.

[53] Yeung AWK Tzvetkov NT Durazzo A Lucarini M Souto EB Santini A. Natural products in diabetes research: quantitative literature analysis. Nat Prod Res. (2020) 1–15. 10.1080/14786419.2020.1821019 33025819

[54] Hu FB Li TY Colditz GA Willett WC Manson JE. Television watching and other sedentary behaviors in relation to risk of obesity and type 2 diabetes mellitus in women. Jama-J Am Med Assoc. (2003) 289:1785–91. 10.1001/jama.289.14.1785 12684356

[55] Cecchini M Sassi F Lauer JA Lee YY Guajardo-Barron V Chisholm D. Chronic Diseases: Chronic Diseases and Development 3 Tackling of unhealthy diets, physical inactivity, and obesity: health effects and cost-effectiveness. Lancet. (2010) 376:1775–84. 10.1016/S0140-6736(10)61514-0 21074255

[56] Kohl HW Craig CL Lambert EV Inoue S Alkandari JR Leetongin G. The pandemic of physical inactivity: global action for public health. Lancet. (2012) 380:294–305. 10.1016/S0140-6736(12)60898-8 22818941

[57] Hruby A Hu FB. The epidemiology of obesity: a big picture. Pharmacoeconomics. (2015) 33:673–89. 10.1007/s40273-014-0243-x 25471927 PMC4859313

[58] Miguet M Fearnbach NS Metz L Khammassi M Julian V Cardenoux C. Effect of HIIT versus MICT on body composition and energy intake in dietary restrained and unrestrained adolescents with obesity. Appl Physiol Nutr Metab. (2020) 45:437–45. 10.1139/apnm-2019-0160 31505120

[59] Su L Fu J Sun S Zhao G Cheng W Dou C. Effects of HIIT and MICT on cardiovascular risk factors in adults with overweight and/or obesity: a meta-analysis. PLoS ONE. (2019) 14:e0210644. 10.1371/journal.pone.0210644 30689632 PMC6349321

[60] Gross M Swensen T King D. Nonconsecutive-versus consecutive-day high-intensity interval training in cyclists. Med Sci Sport Exer. (2007) 39:1666–71. 10.1249/mss.0b013e3180cac209 17805101

[61] Gibala MJ Little JP van Essen M Wilkin GP Burgomaster KA Safdar A. Short-term sprint interval versus traditional endurance training: similar initial adaptations in human skeletal muscle and exercise performance. J Physiol. (2006) 575(Pt 3):901–11. 10.1113/jphysiol.2006.112094 16825308 PMC1995688

[62] Garcia-Hermoso A Cerrillo-Urbina AJ Herrera-Valenzuela T Cristi-Montero C Saavedra JM Martinez-Vizcaino V. Is high-intensity interval training more effective on improving cardiometabolic risk and aerobic capacity than other forms of exercise in overweight and obese youth? A meta-analysis. Obes Rev. (2016) 17:531–40. 10.1111/obr.12395 26948135

[63] Thivel D Masurier J Baquet G Timmons BW Pereira B Berthoin S. High-intensity interval training in overweight and obese children and adolescents: systematic review and meta-analysis. J Sports Med Phys Fitness. (2019) 59:310–24. 10.23736/S0022-4707.18.08075-1 29589408

[64] Dias KA Ingul CB Tjonna AE Keating SE Gomersall SR Follestad T. Effect of high-intensity interval training on fitness, fat mass and cardiometabolic biomarkers in children with obesity: a randomised controlled trial. Sports Med. (2018) 48:733–46. 10.1007/s40279-017-0777-0 28853029

[65] Kujach S Byun K Hyodo K Suwabe K Fukuie T Laskowski R. A transferable high-intensity intermittent exercise improves executive performance in association with dorsolateral prefrontal activation in young adults. Neuroimage. (2018) 169:117–25. 10.1016/j.neuroimage.2017.12.003 29203453

[66] Sanderson WC Scherbov S. Average remaining lifetimes can increase as human populations age. Nature. (2005) 435:811–3. 10.1038/nature03593 15944703

[67] Lutz W Sanderson W Scherbov S. The coming acceleration of global population ageing. Nature. (2008) 451:716–9. 10.1038/nature06516 18204438

[68] Seldeen KL Lasky G Leiker MM Pang M Personius KE Troen BR. High intensity interval training improves physical performance and frailty in aged mice. J Gerontol A Biol Sci Med Sci. (2018) 73:429–37. 10.1093/gerona/glx120 28633487

[69] Hwang CL Lim J Yoo JK Kim HK Hwang MH Handberg EM. Effect of all-extremity high-intensity interval training vs. moderate-intensity continuous training on aerobic fitness in middle-aged and older adults with type 2 diabetes: a randomized controlled trial. Exp Gerontol. (2019) 116:46–53. 10.1016/j.exger.2018.12.013 30576716 PMC6404965

[70] Jimenez-Garcia JD Martinez-Amat A De la Torre-Cruz MJ Fabrega-Cuadros R Cruz-Diaz D Aibar-Almazan A. Suspension training HIIT improves gait speed, strength and quality of life in older adults. Int J Sports Med. (2019) 40:116–24. 10.1055/a-0787-1548 30605922

[71] Kapoor E Collazo-Clavell ML Faubion SS. Weight gain in women at midlife: a concise review of the pathophysiology and strategies for management. Mayo Clin Proc. (2017) 92:1552–8. 10.1016/j.mayocp.2017.08.004 28982486

[72] Lizcano F Guzman G. Estrogen deficiency and the origin of obesity during menopause. Biomed Res Int. (2014) 2014:757461. 10.1155/2014/757461 24734243 PMC3964739

[73] Dolin CD Kominiarek MA. Pregnancy in women with obesity. Obstet Gynecol Clin North Am. (2018) 45:217–32. 10.1016/j.ogc.2018.01.005 29747727

[74] Dupuit M Rance M Morel C Bouillon P Pereira B Bonnet A. Moderate-intensity continuous training or high-intensity interval training with or without resistance training for altering body composition in postmenopausal women. Med Sci Sports Exerc. (2020) 52:736–45. 10.1249/MSS.0000000000002162 31524825

[75] Jabbour G Iancu HD. Comparison of performance and health indicators between perimenopausal and postmenopausal obese women: the effect of high-intensity interval training (HIIT). Menopause. (2020) 28:50–7. 10.1097/GME.0000000000001654 32898025

[76] Steckling FM Farinha JB Figueiredo FDC Santos DLD Bresciani G Kretzmann NA. High-intensity interval training improves inflammatory and adipokine profiles in postmenopausal women with metabolic syndrome. Arch Physiol Biochem. (2019) 125:85–91. 10.1080/13813455.2018.1437750 29431478

[77] Dupuit M Maillard F Pereira B Marquezi ML Lancha AH Jr.. Effect of high intensity interval training on body composition in women before and after menopause: a meta-analysis. Exp Physiol. (2020) 105:1470–90. 10.1113/EP088654 32613697

[78] Cavalot F Petrelli A Traversa M Bonomo K Fiora E Conti M. Postprandial blood glucose is a stronger predictor of cardiovascular events than fasting blood glucose in type 2 diabetes mellitus, particularly in women: lessons from the San Luigi Gonzaga Diabetes Study. J Clin Endocrinol Metab. (2006) 91:813–9. 10.1210/jc.2005-1005 16352690

[79] Cavalot F Pagliarino A Valle M Di Martino L Bonomo K Massucco P. Postprandial blood glucose predicts cardiovascular events and all-cause mortality in type 2 diabetes in a 14-year follow-up: lessons from the San Luigi Gonzaga Diabetes Study. Diabetes Care. (2011) 34:2237–43. 10.2337/dc10-2414 21949221 PMC3177732

[80] Francois ME Baldi JC Manning PJ Lucas SJ Hawley JA Williams MJ. 'Exercise snacks' before meals: a novel strategy to improve glycaemic control in individuals with insulin resistance. Diabetologia. (2014) 57:1437–45. 10.1007/s00125-014-3244-6 24817675

[81] Karstoft K Winding K Knudsen SH Nielsen JS Thomsen C Pedersen BK. The effects of free-living interval-walking training on glycemic control, body composition, and physical fitness in type 2 diabetic patients: a randomized, controlled trial. Diabetes Care. (2013) 36:228–36. 10.2337/dc12-0658 23002086 PMC3554285

[82] Little JP Jung ME Wright AE Wright W Manders RJ. Effects of high-intensity interval exercise versus continuous moderate-intensity exercise on postprandial glycemic control assessed by continuous glucose monitoring in obese adults. Appl Physiol Nutr Metab. (2014) 39:835–41. 10.1139/apnm-2013-0512 24773254

[83] Madsen SM Thorup AC Overgaard K Jeppesen PB. High intensity interval training improves glycaemic control and pancreatic beta cell function of type 2 diabetes patients. PLoS ONE. (2015) 10:e0133286. 10.1371/journal.pone.0133286 26258597 PMC4530878

[84] Cassidy S Thoma C Hallsworth K Parikh J Hollingsworth KG Taylor R. High intensity intermittent exercise improves cardiac structure and function and reduces liver fat in patients with type 2 diabetes: a randomised controlled trial. Diabetologia. (2016) 59:56–66. 10.1007/s00125-015-3741-2 26350611 PMC4670457

[85] Maillard F Rousset S Pereira B Traore A de Pradel Del Amaze P Boirie Y. High-intensity interval training reduces abdominal fat mass in postmenopausal women with type 2 diabetes. Diabetes Metab. (2016) 42:433–41. 10.1016/j.diabet.2016.07.031 27567125

[86] Jelleyman C Yates T O'Donovan G Gray LJ King JA Khunti K. The effects of high-intensity interval training on glucose regulation and insulin resistance: a meta-analysis. Obes Rev. (2015) 16:942–61. 10.1111/obr.12317 26481101

[87] He J Whelton PK. Epidemiology and prevention of hypertension. Med Clin N Am. (1997) 81:1077–97. 10.1016/S0025-7125(05)70568-X 9308599

[88] Kearney PM Whelton M Reynolds K Muntner P Whelton PK He J. Global burden of hypertension: analysis of worldwide data. Lancet. (2005) 365:217–23. 10.1016/S0140-6736(05)17741-1 15652604

[89] James PA Oparil S Carter BL Cushman WC Dennison-Himmelfarb C Handler J. 2014 evidence-based guideline for the management of high blood pressure in adults: report from the panel members appointed to the Eighth Joint National Committee (JNC 8). JAMA. (2014) 311:507–20. 10.1001/jama.2013.284427 24352797

[90] Pescatello LS MacDonald HV Lamberti L Johnson BT. Exercise for hypertension: a prescription update integrating existing recommendations with emerging research. Curr Hypertens Rep. (2015) 17:87. 10.1007/s11906-015-0600-y 26423529 PMC4589552

[91] Pescatello LS MacDonald HV Ash GI Lamberti LM Farquhar WB Arena R. Assessing the existing professional exercise recommendations for hypertension: a review and recommendations for future research priorities. Mayo Clin Proc. (2015) 90:801–12. 10.1016/j.mayocp.2015.04.008 26046413

[92] Costa EC Hay JL Kehler DS Boreskie KF Arora RC Umpierre D. Effects of high-intensity interval training versus moderate-intensity continuous training on blood pressure in adults with pre- to established hypertension: a systematic review and meta-analysis of randomized trials. Sports Med. (2018) 48:2127–42. 10.1007/s40279-018-0944-y 29949110

[93] Sharman JE Smart NA Coombes JS Stowasser M. Exercise and sport science australia position stand update on exercise and hypertension. J Hum Hypertens. (2019) 33:837–43. 10.1038/s41371-019-0266-z 31582784

[94] Lozano R Naghavi M Foreman K Lim S Shibuya K Aboyans V. Global and regional mortality from 235 causes of death for 20 age groups in 1990 and 2010: a systematic analysis for the Global Burden of Disease Study 2010. Lancet. (2012) 380:2095–128. 10.1016/S0140-6736(12)61728-0 23245604 PMC10790329

[95] Gordon NF Gulanick M Costa F Fletcher G Franklin BA Roth EJ. Physical activity and exercise recommendations for stroke survivors: an American Heart Association scientific statement from the council on clinical cardiology, subcommittee on exercise, cardiac rehabilitation, and prevention; the council on cardiovascular nursing; the council on nutrition, physical activity, and metabolism; and the stroke council. Circulation. (2004) 109:2031–41. 10.1161/01.CIR.0000126280.65777.A4 15117863

[96] Billinger SA Arena R Bernhardt J Eng JJ Franklin BA Johnson CM. Physical activity and exercise recommendations for stroke survivors: a statement for healthcare professionals from the American Heart Association/American Stroke Association. Stroke. (2014) 45:2532–53. 10.1161/STR.0000000000000022 24846875

[97] Hornby TG Straube DS Kinnaird CR Holleran CL Echauz AJ Rodriguez KS. Importance of specificity, amount, and intensity of locomotor training to improve ambulatory function in patients poststroke. Top Stroke Rehabil. (2011) 18:293–307. 10.1310/tsr1804-293 21914594

[98] Boyne P Dunning K Carl D Gerson M Khoury J Kissela B. High-intensity interval training in stroke rehabilitation. Top Stroke Rehabil. (2013) 20:317–30. 10.1310/tsr2004-317 23893831

[99] Gjellesvik TI Becker F Tjonna AE Indredavik B Nilsen H Brurok B. Effects of high-intensity interval training after stroke (the HIIT-stroke study): a multicenter randomized controlled trial. Arch Phys Med Rehabil. (2020) 101:939–47. 10.1016/j.apmr.2020.02.006 32145280

[100] Murphy TH Corbett D. Plasticity during stroke recovery: from synapse to behaviour. Nat Rev Neurosci. (2009) 10:861–72. 10.1038/nrn2735 19888284

[101] Dayan E Cohen LG. Neuroplasticity subserving motor skill learning. Neuron. (2011) 72:443–54. 10.1016/j.neuron.2011.10.008 22078504 PMC3217208

[102] Pin-Barre C Constans A Brisswalter J Pellegrino C Laurin J. Effects of high- versus moderate-intensity training on neuroplasticity and functional recovery after focal ischemia. Stroke. (2017) 48:2855–64. 10.1161/STROKEAHA.117.017962 28904232

[103] Luo L Li C Deng Y Wang Y Meng P Wang Q. High-intensity interval training on neuroplasticity, balance between brain-derived neurotrophic factor and precursor brain-derived neurotrophic factor in poststroke depression rats. J Stroke Cerebrovasc Dis. (2019) 28:672–82. 10.1016/j.jstrokecerebrovasdis.2018.11.009 30503681

[104] Pin-Barre C Hugues N Constans A Berton E Pellegrino C Laurin J. Effects of different high-intensity interval training regimens on endurance and neuroplasticity after cerebral ischemia. Stroke. (2021) 52:1109–14. 10.1161/STROKEAHA.120.031873 33517700

[105] Pugh JK Faulkner SH Turner MC Nimmo MA. Satellite cell response to concurrent resistance exercise and high-intensity interval training in sedentary, overweight/obese, middle-aged individuals. Eur J Appl Physiol. (2018) 118:225–38. 10.1007/s00421-017-3721-y 29071380 PMC5767196

[106] Li FH Sun L Zhu M Li T Gao HE Wu DS. Beneficial alterations in body composition, physical performance, oxidative stress, inflammatory markers, and adipocytokines induced by long-term high-intensity interval training in an aged rat model. Exp Gerontol. (2018) 113:150–62. 10.1016/j.exger.2018.10.006 30308288

[107] Li FH Sun L Wu DS Gao HE Min Z. Proteomics-based identification of different training adaptations of aged skeletal muscle following long-term high-intensity interval and moderate-intensity continuous training in aged rats. Aging. (2019) 11:4159–82. 10.18632/aging.102044 31241467 PMC11623340

[108] Marzuca-Nassr GN Artigas-Arias M Olea MA SanMartin-Calisto Y Huard N Duran-Vejar F. High-intensity interval training on body composition, functional capacity and biochemical markers in healthy young versus older people. Exp Gerontol. (2020) 141:111096. 10.1016/j.exger.2020.111096 32971179

[109] Leuchtmann AB Mueller SM Aguayo D Petersen JA Ligon-Auer M Fluck M. Resistance training preserves high-intensity interval training induced improvements in skeletal muscle capillarization of healthy old men: a randomized controlled trial. Sci Rep. (2020) 10:6578. 10.1038/s41598-020-63490-x 32313031 PMC7171189

[110] Gibala MJ Jones AM. Physiological and performance adaptations to high-intensity interval training. Nestle Nutr Inst Workshop Ser. (2013) 76:51–60. 10.1159/000350256 23899754

[111] Burgomaster KA Hughes SC Heigenhauser GJ Bradwell SN Gibala MJ. Six sessions of sprint interval training increases muscle oxidative potential and cycle endurance capacity in humans. J Appl Physiol. (2005) 98:1985–90. 10.1152/japplphysiol.01095.2004 15705728

[112] Barry JC Simtchouk S Durrer C Jung ME Little JP. Short-term exercise training alters leukocyte chemokine receptors in obese adults. Med Sci Sports Exerc. (2017) 49:1631–40. 10.1249/MSS.0000000000001261 28319586

[113] Pedersen BK Fischer CP. Beneficial health effects of exercise–the role of IL-6 as a myokine. Trends Pharmacol Sci. (2007) 28:152–6. 10.1016/j.tips.2007.02.002 17331593

[114] Petersen AM Pedersen BK. The anti-inflammatory effect of exercise. J Appl Physiol. (2005) 98:1154–62. 10.1152/japplphysiol.00164.2004 15772055

[115] Pedersen BK Febbraio MA. Muscle as an endocrine organ: focus on muscle-derived interleukin-6. Physiol Rev. (2008) 88:1379–406. 10.1152/physrev.90100.2007 18923185

[116] Steensberg A Fischer CP Keller C Moller K Pedersen BK. IL-6 enhances plasma IL-1ra, IL-10, and cortisol in humans. Am J Physiol Endocrinol Metab. (2003) 285:E433–7. 10.1152/ajpendo.00074.2003 12857678

[117] Barry JC Simtchouk S Durrer C Jung ME Mui AL Little JP. Short-term exercise training reduces anti-inflammatory action of interleukin-10 in adults with obesity. Cytokine. (2018) 111:460–9. 10.1016/j.cyto.2018.05.035 29885989

[118] Lee JS Boafo A Greenham S Longmuir PE. The effect of high-intensity interval training on inhibitory control in adolescents hospitalized for a mental illness. Ment Health Phys Act. (2019) 17:100298. 10.1016/j.mhpa.2019.100298

[119] Martland R Mondelli V Gaughran F Stubbs B. Can high intensity interval training improve health outcomes among people with mental illness? A systematic review and preliminary meta-analysis of intervention studies across a range of mental illnesses. J Affect Disord. (2020) 263:629–60. 10.1016/j.jad.2019.11.039 31780128

[120] Korman N Armour M Chapman J Rosenbaum S Kisely S Suetani S. High Intensity Interval training (HIIT) for people with severe mental illness: a systematic review & meta-analysis of intervention studies- considering diverse approaches for mental and physical recovery. Psychiat Res. (2020) 284:112601. 10.1016/j.psychres.2019.112601 31883740

[121] Leahy AA Mavilidi MF Smith JJ Hillman CH Eather N Barker D. Review of high-intensity interval training for cognitive and mental health in youth. Med Sci Sports Exerc. (2020) 52:2224–34. 10.1249/MSS.0000000000002359 32301856

[122] Jimenez-Pavon D Carbonell-Baeza A Lavie CJ. Physical exercise as therapy to fight against the mental and physical consequences of COVID-19 quarantine: special focus in older people. Prog Cardiovasc Dis. (2020) 63:386–8. 10.1016/j.pcad.2020.03.009 32220590 PMC7118448

[123] Li Y Liu D Wu H. HIIT: a potential rehabilitation treatment in COVID-19 pneumonia with heart disease. Int J Cardiol. (2020) 320:183. 10.1016/j.ijcard.2020.07.030 32721412 PMC7833490

