# Supplementary figures and images for: Bibliometric Review to Explore Emerging High-Intensity Interval Training in Health Promotion: A New Century Picture
Source: Front Public Health. 2021 Jul 23;9:697633. doi: 10.3389/fpubh.2021.697633 (PMC8342813; doi:10.3389/fpubh.2021.697633)

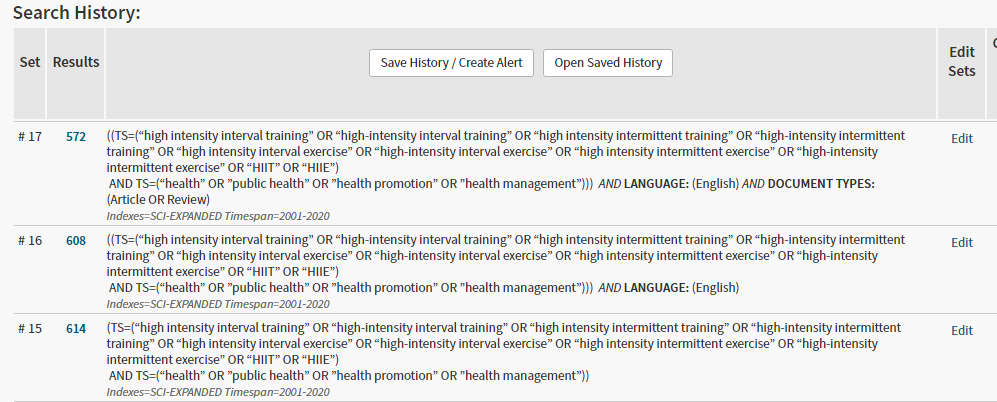

Supplement: Supplementary file 1 [file Data_Sheet_1.ZIP › search history.png]
